# Correlation between nova volume flow rate and TOF signal intensity ratio: value in unilateral internal carotid artery occlusion

**DOI:** 10.1007/s11547-024-01917-5

**Published:** 2024-11-12

**Authors:** Fabian Wolf, Elisa Colombo, Tilman Schubert, Lara Maria Höbner, Susanne Wegener, Jorn Fierstra, Martina Sebök, Bas van Niftrik, Andreas Luft, Luca Regli, Giuseppe Esposito

**Affiliations:** 1 Department of Neurosurgery and Clinical Neuroscience Center, Universität Zürich, Universitätsspital Zürich, Frauenklinikstrasse 10, 8091 Zurich, ZH Switzerland; 2 Department of Neuroradiology, Universitätsspital Zürich, Zurich, ZH Switzerland; 3 Department of Neurology, Universitätsspital Zürich, Zurich, ZH Switzerland

**Keywords:** Intracranial atherosclerosis, ICA occlusion, Cerebral perfusion, Quantitative magnetic resonance imaging, NOVA, Volume flow rate, TOF

## Abstract

**Background and purposes:**

Non-invasive optimal vessel analysis quantitative magnetic resonance angiography (NOVA-QMRA) has emerged as a valuable tool to characterize cerebral hemodynamics in intracranial atherosclerotic disease (ICAD). Our aim was to explore the eventual correlation between volume flow rate (VFR) measured via NOVA-QMRA and signal intensity ratio (SIR) of time-of-flight (TOF) MRA in M1- and P2-segments bilaterally in patients with unilateral internal carotid artery (ICA) occlusion.

**Materials and methods:**

Patients with acute, subacute or chronic unilaterall ICA occlusion receiving NOVA-QMRA between June 2019 and June 2021 were retrospectively included. In bilateral M1- and P2-segments VFR was assessed by means of NOVA-QMRA and a region of interest (ROI) was selected to measure TOF SIR. A correlation between TOF SIR and VFR was tested by means of Pearson correlation coefficient. Mean difference of TOF SIR and VFR between ipsilateral (to occluded ICA) and contralateral M1- and P2-segments was analyzed using a two-sided Welch’s t test.

**Results:**

Fifty-five patients with unilateral ICA occlusion were included (acute: 28; subacute: 8; chronic: 19). Both ipsilateral (r = 0.536, p < 0.001) and contralateral (r = 0.757, *p* < 0.001) TOF SIR correlated significantly with NOVA VFR. This observation proved especially true for patients with chronic ICA occlusion. Both VFR (165.18 vs 110.60, *p* < 0.001) and TOF SIR (4.96 vs 2.70, *p* < 0.001) were higher in contralateral than ipsilateral M1-segments; whereas, the contrary was observed for P2-segments (VFR 72.35 vs 102.12, *p* < 0.001, TOF SIR 2.87 vs 3.39, *p* = 0.016).

**Conclusion:**

The study results showed that TOF SIR significantly correlated with phase-contrast derived flow volume in patients with symptomatic ICA occlusion. This correlation remains the same regardless of the stage of the ischemic stroke (acute vs subacute vs chronic). Furthermore, significantly high VFR and TOF SIR in ipsilateral P2-segments may provide evidence of leptomeningeal collateralization in acute patients. Standardly performed TOF SIR Sequences might be of help for a qualitative evaluation of the flow in M1- and P2-segments in patients with unilateral ICA occlusions. NOVA QMRA allows precise quantitative measurements of the flow in cerebral vessels.

**Graphical abstract:**

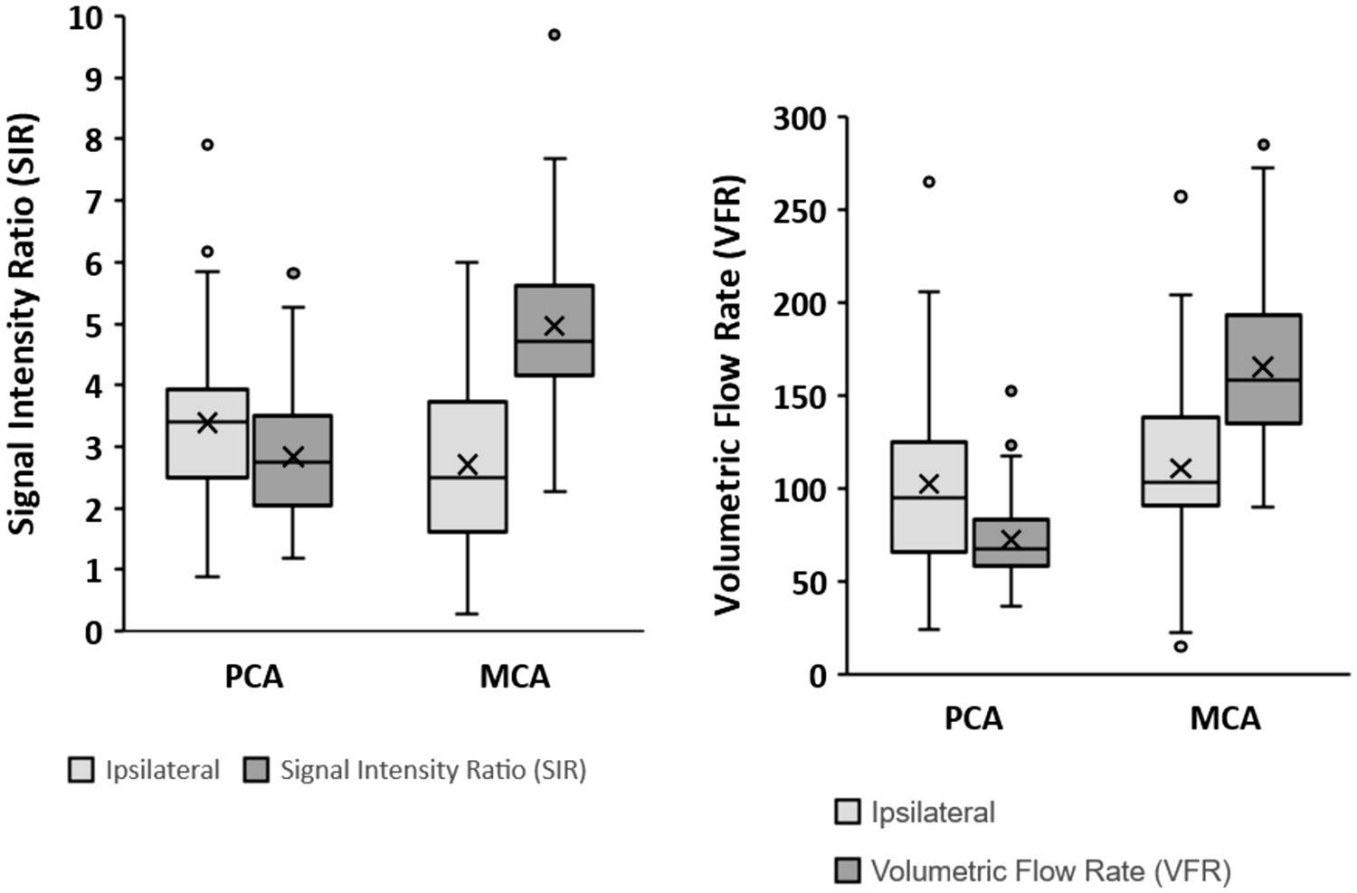

## Introduction

With the improvement in cerebrovascular imaging techniques, the use of non-invasive MR angiography has become of increasing importance for an accurate evaluation and risk assessment of patients presenting with ICA occlusion [1]. Intracranial atherosclerotic disease (ICAD) is one of the most relevant causes of acute ischemic stroke worldwide [2, 3]. In the settings of ICAD, ICA occlusion contributes to 10% to 20% of strokes or transient ischemic attacks [4, 5].

**Fig. 1:**
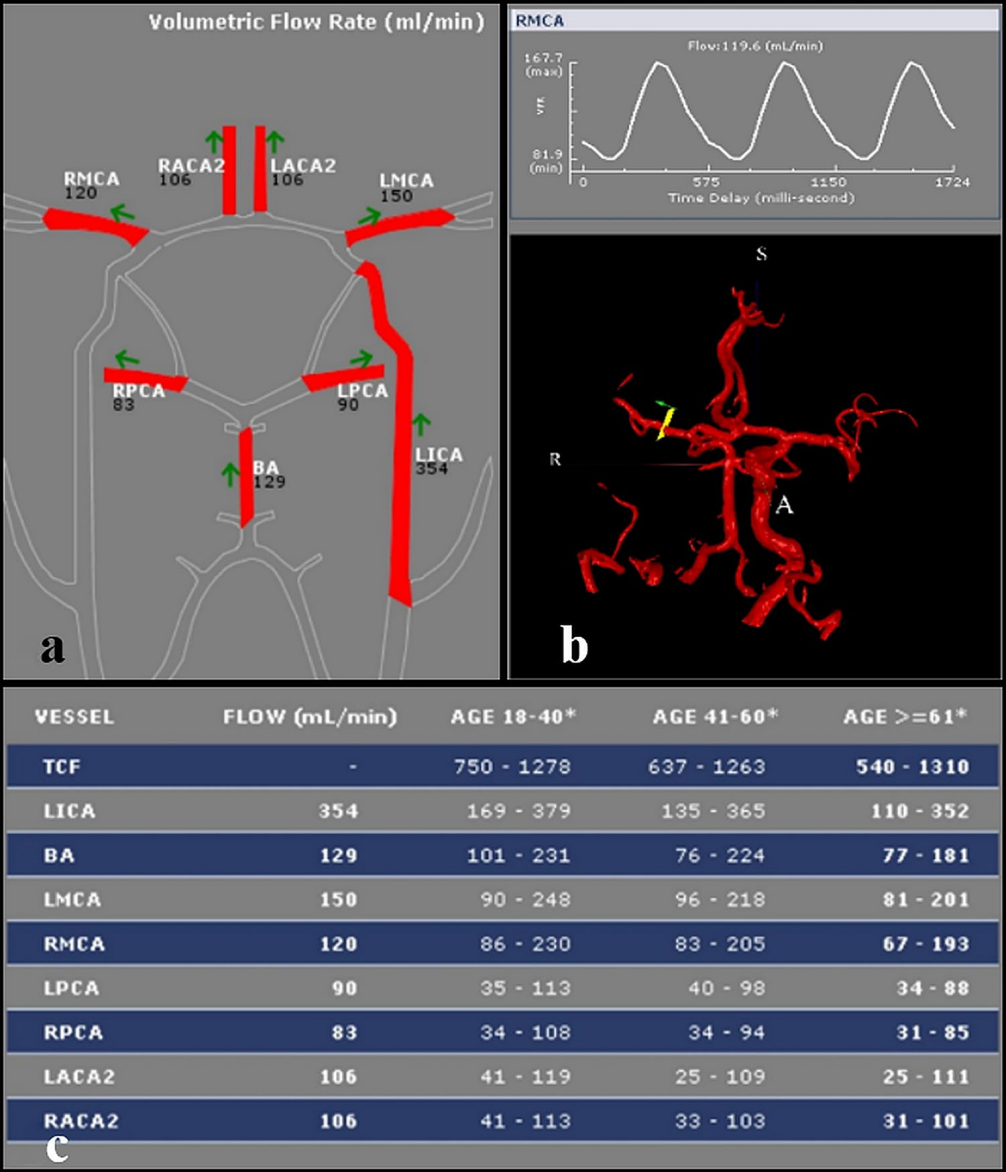
NOVA-QMRA map of a 77-years old patient presenting with a right MCA stroke due to an acute occlusion of the ipsilateral ICA (1a). A table showing the VFRs of the patient and the range of each major intracranial artery’s VFR according to age (1c) accompanies every map. Furthermore, a graphic representation of how the VFR was evaluated for each artery with the associated curve and the direction (anterograde or retrograde) are also provided (1b). NOVA-QMRA: non-invasive optimal vessel analysis quantitative magnetic resonance angiography; MCA: middle cerebral artery; ICA: internal cerebral artery; VFR: volume flow rate

Non-invasive optimal vessel analysis quantitative magnetic resonance angiography (NOVA-QMRA) uses TOF and Phase Contrast (PC) MRA sequences for precise quantitative (in ml/min) measurement of blood flow in the cerebral vessels [6–13]. This way it also allows reliable quantification of cerebral collateral pathways in cases of large vessel occlusion [14–16] (Fig. 1). In the settings of MRA, the use of TOF-sequences has been established for the study of stroke patients [17, 18] and is routinely applied to detect and assess large artery stenosis or occlusion [19]. An attempt to correlate TOF signal intensity and VFR assessed via NOVA-QMRA has not been done yet.

In the present study, we explored this correlation in patients with unilateral ICA occlusion: VFR (via NOVA-QMRA) and TOF SIR (signal intensity ratio) were measured in M1-segments of the middle cerebral artery (MCA) and P2-segments of the posterior cerebral artery (PCA) bilaterally (ipsilateral and contralateral to the occluded ICA).

The purpose of this analysis is to investigate the value of qualitative assessment of SIR in TOF MRA in comparison with quantitative measurement of VFR by NOVA-QMRA in patients with acute, subacute and chronic unilateral ICA occlusion. We hypothesize that a correlation between SIR and VFR could be valuable for rapidly and qualitatively assess blood flow in patients with symptomatic ICA occlusion. SIR could represent a surrogate for VFR in institutions without access to QMRA.

## Materials and methods

Patients who received a NOVA-QMRA because of unilateral ICA steno-occlusive disease between June 2019 and June 2021 at the Neuroscience Clinical Center of the University Hospital Zurich were retrospectively identified. Patients who met the following criteria were included in the study: 1) Patient older than 18; 2) symptomatic occlusion of one ICA; 3) Contralateral ICA patency or stenosis ≤ 50%. The study was approved by the local Ethics Committee and performed in accordance with the Declaration of Helsinki. Patients provided informed consent for the use of personal data for scientific purposes at the time of admission.

Carotid artery occlusion was diagnosed by means of neuroimaging, i.e., CTA and/or MRA, as well as by Doppler ultrasound performed by an experienced neurologist; and rated according to the North American Symptomatic Carotid Endarterectomy Trial (NASCET) criteria as ‘no flow’ [20].

QMRA examinations were performed on a 3 T MRI scanner (Magnetom Skyre, Siemens Healthineers, Erlangen, Germany). Depending on the time between ICA occlusion symptoms and MRI protocol the patients were divided into three subgroups: Acute) NOVA-QMRA performed less than one week after symptoms; Subacute) NOVA-QMRA performed one to six weeks after symptoms; Chronic) NOVA-QMRA performed more than six weeks after symptoms.

### MR vessel imaging techniques

**Fig. 2:**
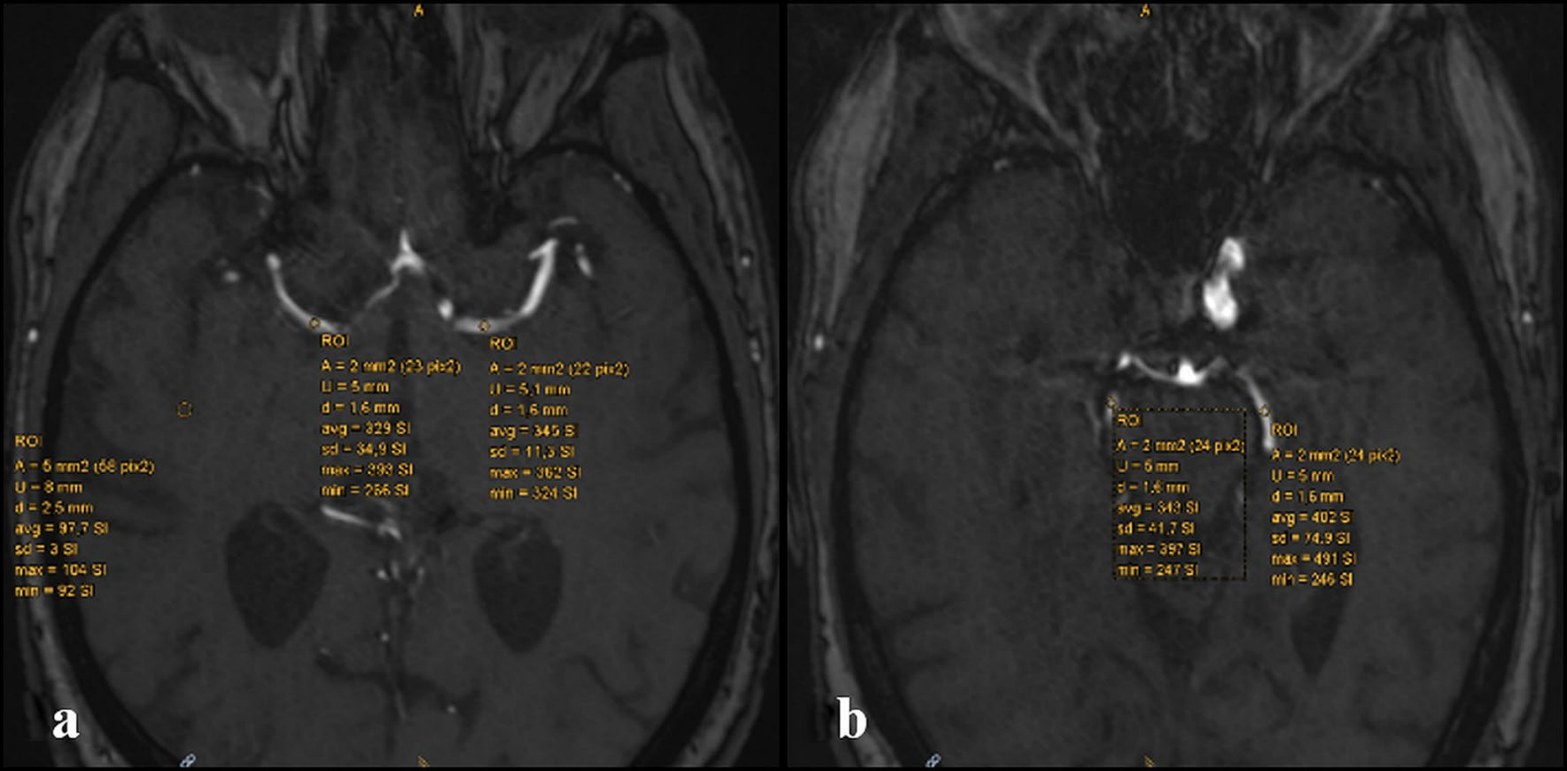
Starting from a 3D-MRA-TOF, a slice showing the bilateral MCA.M1 segments and another with bilateral PCA-P2-segments are chosen. For each artery, a ROI of 2mm^2^ is selected (2a-b). To adjust the measured values for variable background noise a reference signal intensity is measured in a 5mm2 ROI in avascular white matter (2a). 3D: three-dimensional; MRA: magnetic resonance imaging; TOF: time-of-flight; ROI: region of interest

Axial native 3D TOF sequence images were used to gather the SI of M1- and P2-segment as well as background intensity. For each patient a region of interest (ROI) with an area of two square millimeters was selected over the vessel. SI was measured for bilateral M1- and P2-segments (Fig. 2a–b). To adjust the measured values for variable background noise a reference signal intensity was measured in a 5mm^2^ ROI in avascular white matter (Fig. 2a). The background ROI laid in the same slice segment as the examined vessels, following the technique described by previous literature [21, 22]. SI ratio (SIR) was calculated as followed: Mean arterial signal intensity / mean background intensity.

When performing a NOVA QMRA the TOF MRA sequence is acquired first. TOF images are sent to a remote workstation (NOVA TECH PC, VasSol Inc.) where a 3D reconstruction of vessel surfaces is rendered. The examiner, a NOVA-certified specialist from the Department of Neuroradiology and/or Neurosurgery, determines the vessels of interest (where the flow has to be measured) and sets a perpendicular plane to the selected arteries. This information is returned to the MRI scanner and subsequent PC MRA sequences are performed to measure the arterial VFR in the selected planes [13].

### Statistical analysis

All statistical analyses were performed using R Studio (RStudio, PBC, Boston). Data were presented as numbers and percentages, means and standard deviations (SD). A simple Pearson correlation coefficient for the association of VFR and TOF SIR was calculated. To compare ipsilateral and contralateral VFR and TOF SIR a two-sided Welch’s t test was performed.

## Results

*Study population* (Table 1).

**Table 1:** Patient demographics and cause of ICA occlusion

Age	63.4 (± 13, 29–89)
Sex	70% male (n = 39)
Total	55
TOAST 1	30 (55%)
TOAST 2	1 (2%)
TOAST 3	0 (0%)
TOAST 4	4 (7%)
TOAST 5	20 (36%)

TOAST, trial of ORG 10172 in acute stroke treatment

Between June 2019 and June 2021, 120 patients with ICA stenosis or occlusion received a NOVA-QMRA in our Institution. Fifty-six patients met the inclusion criteria: 28 (51%) with acute, 8 (14%) with subacute and 19 (35%) with chronic ICA occlusion. The mean age of the cohort was 63.4 years (± 13, 29–89; SD, range). Thirty-nine (71%) patients were male. The etiology of ICA occlusion was categorized according to the TOAST classification [23]. The most common cause for unilateral ICA occlusion was large artery atherosclerosis (n = 30, 55%), followed by events of undetermined etiology (n = 20, 36%). Four patients (7%) presented occlusion of other determined etiology, while only one case (2%) of cardioembolism was reported. The other sixty-five patients were excluded for following reason: 58 presented with ICA stenosis < 100%, 7 with a contralateral ICA stenosis greater than 50%.

*Statistical analysis* (Table 2).

**Table 2:** Comparative analysis of correlation between VFR and TOF SIR

		Ipsilateral	Contralateral
All	Total	R = 0.536, *p* < 0.001, n = 93	R = 0.757, *p* < 0.001, n = 101
	M1-MCA	R = 0.592, *p* < 0.001, n = 43	R = 0.556, *p* < 0.001, n = 50
	P2-PCA	R = 0.608, *p* < 0.001, n = 50	R = 0.451, *p* < 0.001, n = 51
Acute	Total	R = 0.504, *p* < 0.001, n = 49	R = 0.785, *p* < 0.001, n = 51
	M1-MCA	R = 0.704, *p* < 0.001, n = 23	R = 0.622, *p* < 0.001, n = 25
	P2-PCA	R = 0.437, *p* = 0.025, n = 26	R = 0.318, *p* = 0.11, n = 26
Subacute	Total	R = 0.407, *p* = 0.13, n = 15	R = 0.677, *p* < 0.005, n = 16
	M1-MCA	R = − 0.360, *p* = 0.432, n = 7	R = − 0.295, *p* = 0.48, n = 8
	P2-PCA	R = 0.884, *p* < 0.005, n = 8	R = 0.804, *p* < 0.05, n = 8
Chronic	Total	R = 0.600, *p* < 0.001, n = 29	R = 0.787, *p* < 0.001, n = 34
	M1-MCA	R = 0.543, *p* = 0.05, n = 13	R = 0.629, *p* < 0.01, n = 17
	P2-PCA	R = 0.669, *p* < 0.005, n = 16	R = 0.372, *p* = 0.14, n = 17

M1-MCA, M1 segment of the middle cerebral artery, P2-PCA: P2-segment of the posterior cerebral artery; VFR, volume flow rate; TOF SIR, time-of-flight signal intensity ratio

A significant correlation was observed between TOF SIR and VFR over the entire cohort in both ipsi- and contralateral M1- and P2-segments. M1- and P2-segments contralateral to the occlusion presented a higher correlation (r = 0.757, 95%-CI: 0.660–0.830, *p* < 0.001, n = 101) than ipsilaterally to ICA occlusion (r = 0.536, 95%-CI: 0.373–0.667, *p* < 0.001, n = 93). This observation held true for acute, subacute and chronic patients.

*Acute patients* (NOVA performed within 1 week after symptoms) – > Vessels in this subgroup showed a higher correlation between VFR and TOF SIR on the contralateral side (r = 0.785, 95%-CI: 0.649–0.872, *p* < 0.001, n = 51) than for M1- and P2-segments ipsilaterally to the occlusion (r = 0.504, 95%-CI: 0.259–0.687, *p* > 0.001, n = 49).

*Subacute patients* (NOVA performed 1 to 6 weeks after symptoms)—> In this subgroup, only the contralateral vessels showed a significant correlation (r = 0.677, 95%-CI: 0.272–0.878, *p* < 0.005); while, the correlation of M1- and P2- segments on the ipsilateral side was not statistically significant (r = 0.407, 95%-CI: -0.133–0.760, p = 0.13). Chronic patients (NOVA performed > 6 weeks after symptoms)—> The highest correlation between VFR and TOF SIR was observed in this subgroup. It follows the same pattern, contralateral segments showed a high correlation coefficient of r = 0.787 (95%-CI: 0.611–0.889, p < 0.001); whereas, the ipsilateral segments only presented with a moderate correlation (r = 0.600, 95%-CI: 0.300–0.792, *p* < 0.001).

*Magnitude of TOF SIR and VFR in relation to site of ICA occlusion* (Table 3).

**Table 3:** Mean difference in VFR and TOF SIR between ipsi- and contralateral segments

		Ipsilateral	Contralateral	Mean difference	*p*-value
All	M1-MCA VFR	110.60 (n = 45)	165.18 (n = 50)	54.58 (± 19.63)	< 0.001
	P2-PCA VFR	102.12 (n = 51)	72.35 (n = 51)	− 29.76 (± 14.74)	< 0.001
	M1-MCA TOF SIR	2.70 (n = 48)	4.96 (n = 55)	2.26 (± 0.53)	< 0.001
	P2-PCA TOF SIR	3.39 (n = 54)	2.84 (n = 55)	− 0.55 (± 0.44)	< 0.05
Acute	M1-MCA VFR	119.17 (n = 24)	172.32 (n = 25)	53.15 (± 26.52)	< 0.001
	P2-PCA VFR	107.04 (n = 26)	75.65 (n = 26)	− 31.38 (± 17.97)	< 0.001
	M1-MCA TOF SIR	2.88 (n = 25)	5.41 (n = 28)	2.53 (± 0.76)	< 0.001
	P2-PCA TOF SIR	3.54 (n = 28)	3.10 (n = 28)	− 0.44 (± 0.63)	0.1629
Subacute	M1-MCA VFR	101.43 (n = 7)	140.75 (n = 8)	39.32 (± 37.34)	< 0.05
	P2-PCA VFR	101.25 (n = 8)	69.88 (n = 8)	− 31.38 (± 39.28)	0.106
	M1-MCA TOF SIR	1.67 (n = 7)	4.36 (n = 8)	2.69 (± 0.87)	< 0.001
	P2-PCA TOF SIR	3.37 (n = 8)	2.39 (n = 8)	− 0.98 (± 1.39)	0.155
Chronic	M1-MCA VFR	102.80 (n = 15)	166.18 (n = 17)	63.38 (± 40.22)	< 0.005
	P2-PCA VFR	95.00 (n = 17)	68.47 (n = 17)	− 26.53 (± 33.02)	0.11
	M1-MCA TOF SIR	2.89 (n = 16)	4.55 (n = 19)	1.66 (± 0.93)	< 0.005
	P2-PCA TOF SIR	3.16 (n = 18)	2.65 (n = 19)	− 0.51 (± 0.75)	0.17

M1-MCA, M1 segment of the middle cerebral artery; P2-PCA, P2-segment of the posterior cerebral artery; VFR, volume flow rate; TOF SIR, time-of-flight signal intensity ratio

**Fig. 3:**
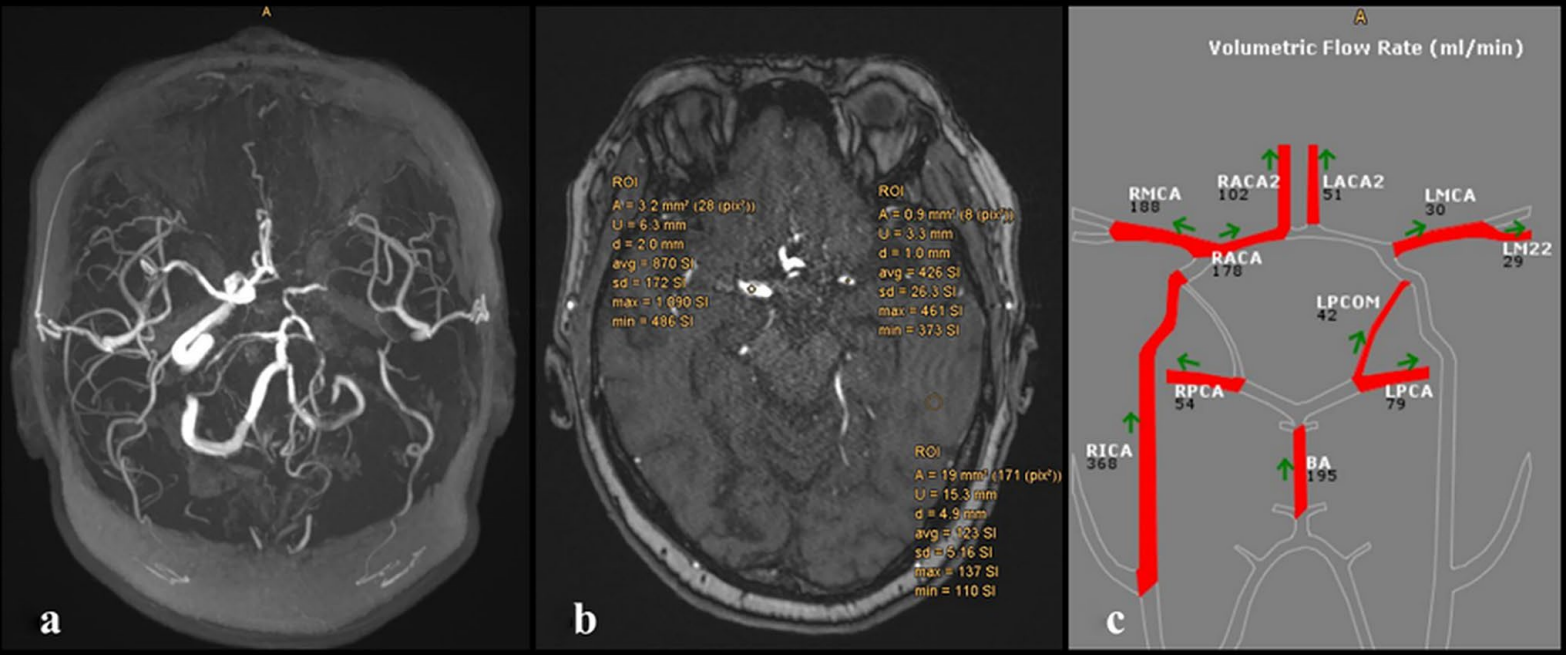
Scatterplots showing the correlation between the magnitude of VFR and TOF SIR in ipsilateral (3a) and contralateral (3b) M1- and P2-segments

The magnitude of VFR and TOF SIR are dependent on the localization of the examined vessel in relation to the ICA occlusion. Throughout the entire study population mean VFR and TOF SIR are significantly higher in contralateral than ipsilateral M1-segments (VFR 165.18 vs 110.60, *p* < 0.001, TOF SIR 4.96 vs 2.70, *p* < 0.001). For P2-segments the data behaves inversely with higher VFR and TOF SIR ipsilateral to the occlusion (VFR 72.35 vs 102.12, *p* < 0.001, TOF SIR 2.84 vs 3.39, *p* = 0.016). This statistical significance holds especially true in patients with acute ICA occlusion. A similar hemodynamic behavior is observed in the subacute and chronic subgroup but with lower levels of significance. Figure 3 demonstrates graphically the correlation between the magnitude of VFR and TOF SIR in ipsilateral (3a) and contralateral (3b) M1- and P2-segments.

## Discussion

The use of QMRA becomes of increasing importance to study ischemic stroke in the context of intracranial atherosclerosis [24]. TOF MRA allows an accurate assessment of the severity of narrowed or occluded intracranial vessels [24]. While NOVA QMRA allows a more comprehensive evaluation of the cerebral flow and hemodynamics (collateralization) in ischemic stroke through non-invasive measurement of VFR [14], it is a highly specific software available in selected neurovascular centers. On the other hand, TOF MRA is accepted as a standard imaging technique in cerebrovascular examination and widely accessible even in regional hospitals. Both imaging modalities are greatly beneficial in the evaluation of large vessel steno-occlusive disease, but, to our knowledge, no record of comparison between NOVA QMRA and MRA TOF is found in recent literature, neither in clinical settings nor with healthy subjects.

This study is the first on in which the correlation between SIR and VFR was evaluated in a cohort of patients presenting with symptomatic unilateral ICA occlusion. The comparison was performed between the M1- and P2-segments ipsi- and contralateral to the occlusion.

The analysis showed a significant correlation between VFR measured via NOVA QMRA and SIR in TOF MRA. The strength of this correlation is moderate in M1- and P2-segments ipsilateral to the occluded ICA; while, the contralateral vessels show strong correlations with R-values greater than 0.7.

Subgroup analysis (acute, subacute and chronic groups) confirmed the significant correlation regardless of time of execution of MR after symptoms. Although this statement holds generally true, further analysis between ipsi- and contralateral M1- and P2-segments in the different subgroups shows few exceptions that need to be discussed (Table 3).

When considering the vessels specifically, in the subgroup of patients with acute ICA occlusions, we observed a weaker correlation between VFR and TOF SIR in the contralateral M1- and P2-segment. The weaker correlation in those specific arteries could be explained by the impaired intracranial hemodynamic following the first occurrence of the symptomatic occlusion [25].

In the subgroup of patient with subacute ICA occlusions, we could not find significant p values in the ipsi- and contralateral M1-segments. This result is most likely due to a disproportionate number of tandem occlusions (3/7, respectively, 3/8) in this subgroup.

The strongest correlation between NOVA VFR and TOF SIR was found in the vessels of patients with chronic ICA occlusion. Probably because this group had the greatest chance to undergo a long-term adaptation and see the re-emergence of a hemodynamic equilibrium after the ICA occlusion [25, 26] (Table 2).

Another important finding regards the difference in hemodynamic behavior between ipsi- and contralateral M1 and P2 in the whole cohort. While M1 VFR and SIR tend to be higher in the contralateral side to the occlusion, the opposite is true for P2 VFR and SIR (Table 3).

The higher VFR and TOF SIR in ipsilateral P2-segments support the concept of previous studies that showed that cerebral perfusion of the ipsilateral hemisphere may be rescued by activation of leptomeningeal collaterals [27].

The present comparative analysis was designed to assess whether TOF SIR could give a rapid qualitative information about flow/perfusion in patients with symptomatic ICA occlusion. TOF SIR does correlate with VFRs of the ipsi- and contralateral M1- and P2-segments, although the correlation is higher on the side contralateral to ICA occlusion.

Although able to show a significant correlation between VFR and TOF SIR in M1- and P2-segments the obtained insights need to be interpreted in the framework of the retrospective nature and small sample size of this study.

Another limiting factor for the accuracy of the correlation between VFR and TOF SIR is the impact of arterial geometry on the SI in TOF MRA imaging. Contrast between vessels and stationary tissue is produced by flow related enhancement, a phenomenon that is highly dependent on perpendicular out of scan plane movement of blood. Arteries staying in the scan plane or even presenting with a backwards flow experience a spin saturation and therefore loose flow related enhancement faster, thus displaying a lower SI than arteries that move out of the scan plane. Furthermore, this study did not investigate further the added value of NOVA-QMRA compared to other advanced imaging modalities which study cerebral perfusion in patients presenting intracranial large vessel occlusions. Indeed this aspect should represent the focus for future research to further validate the use of NOVA in clinical settings. Lastly, in our study population, only a very limited number of patients were identified as having Circle of Willis variants; therefore, no analysis differentiating between a normal and a Circle of Willis showing anatomical variations was possible.

**Fig. 4:**
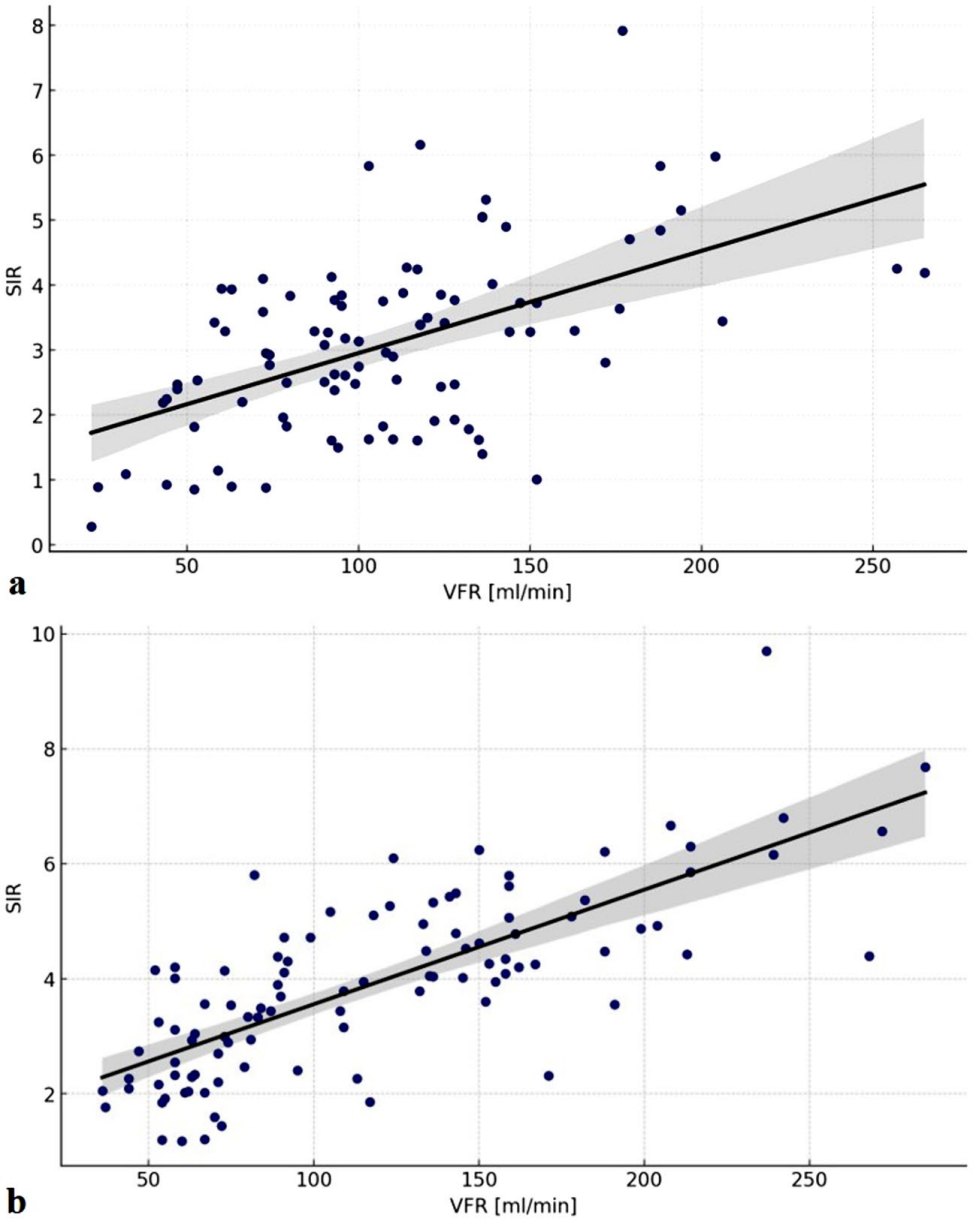
Graphs show the difference between mean SIR and VFR according to examined vessel and side of occluded ICA. ICA: internal carotid artery; SIR: signal intensity ratio; VFR: volume flow rate

Nonetheless, TOF SIR sequences could be valuable for a rapid and qualitative assessment of the flow in M1- and P2-segments in patients with unilateral ICA occlusions; while, NOVA QMRA remains the only technique allowing precise quantitative measurement in ml/min. Figures 1 and 2 display this relationship: Fig. 1 shows symmetrical results of TOF SIR and NOVA VFR in bilateral M1-segments (Fig. 1); whereas, Fig. 3 shows a clear asymmetry of the SIRs in the two M1-segments and consequently different NOVA VFRs (Fig. 4). Higher VFR and TOF signal in ipsilateral P2-segments suggest an activation of the leptomeningeal collateral pattern. The results support the use of TOF SIR as a qualitative surrogate of NOVA-VFR. These findings may promote the referral of patients with symptomatic ICA occlusion to tertiary centers for further flow hemodynamic evaluation.

## Conclusion

TOF SIR significantly correlates with VFR in patients with symptomatic ICA occlusion regardless of the stage of the ischemic stroke (acute vs subacute vs chronic). Significantly higher VFR and TOF SIR in ipsilateral P2-segments may provide evidence of leptomeningeal collateralization. Therefore, TOF SIR could be valuable to provide a qualitative evaluation of M1 and P2 flow; while, NOVA-QMRA remains the only technique allowing precise quantitative measurement in ml/min.

## References

[1] Brinjikji W, Rabinstein AA, Meyer FB, Piepgras DG, Lanzino G (2010) Risk of early carotid endarterectomy for symptomatic carotid stenosis. Stroke 41(10):2186–2190. 10.1161/STROKEAHA.110.590711 20798367 10.1161/STROKEAHA.110.590711

[2] Yaghi S, Rostanski SK, Boehme AK et al (2016) Imaging parameters and recurrent cerebrovascular events in patients with minor stroke or transient ischemic attack. JAMA Neurol 73(5):572–578. 10.1001/jamaneurol.2015.4906 26998948 10.1001/jamaneurol.2015.4906 PMC5022560

[3] Wong LKS (2006) Global burden of intracranial atherosclerosis. Int J Stroke 1(3):158–159. 10.1111/j.1747-4949.2006.00045.x 18706036 10.1111/j.1747-4949.2006.00045.x

[4] Paciaroni M, Caso V, Venti M et al (2005) Outcome in patients with stroke associated with internal carotid artery occlusion. Cerebrovasc Dis 20(2):108–113. 10.1159/000086800 16006758 10.1159/000086800

[5] Yaghi S, Prabhakaran S, Khatri P, Liebeskind DS (2019) Intracranial atherosclerotic disease. Stroke 50(5):1286–1293. 10.1161/STROKEAHA.118.024147 31009344 10.1161/STROKEAHA.118.024147

[6] Amin-Hanjani S, Singh A, Rifai H et al (2013) Combined direct and indirect bypass for moyamoya: quantitative assessment of direct bypass flow over time. Neurosurgery 73(6):962–967. 10.1227/NEU.0000000000000139 23949274 10.1227/NEU.0000000000000139

[7] Shakur SF, Amin-Hanjani S, Bednarski C et al (2015) Intracranial blood flow changes after extracranial carotid artery stenting. Neurosurgery 76(3):330–336. 10.1227/NEU.0000000000000618 25599202 10.1227/NEU.0000000000000618

[8] Esfahani DR, Stevenson M, Moss HE et al (2015) Quantitative magnetic resonance venography is correlated with intravenous pressures before and after venous sinus stenting: implications for treatment and monitoring. Neurosurgery 77(2):254–260. 10.1227/NEU.0000000000000771 25860429 10.1227/NEU.0000000000000771 PMC4506216

[9] Khan N, Lober RM, Ostergren L et al (2017) Measuring cerebral blood flow in moyamoya angiopathy by quantitative magnetic resonance angiography noninvasive optimal vessel analysis. Neurosurgery 81(6):921–927. 10.1093/neuros/nyw122 28204602 10.1093/neuros/nyw122

[10] Shakur SF, Aletich VA, Amin-Hanjani S, Hussein AE, Charbel FT, Alaraj A (2017) Quantitative assessment of parent vessel and distal intracranial hemodynamics following pipeline flow diversion. Interv Neuroradiol 23(1):34–40. 10.1177/1591019916668842 27703060 10.1177/1591019916668842 PMC5305150

[11] Prabhakaran S, Warrior L, Wells KR, Jhaveri MD, Chen M, Lopes DK (2009) The utility of quantitative magnetic resonance angiography in the assessment of intracranial in-stent stenosis. Stroke 40(3):991–993. 10.1161/STROKEAHA.108.522391 19164797 10.1161/STROKEAHA.108.522391

[12] Brunozzi D, Theiss P, Andrews A, Amin-Hanjani S, Charbel FT, Alaraj A (2019) Correlation between laminar wall shear stress and growth of unruptured cerebral aneurysms. In Vivo Assessment World Neurosurg 131:e599–e605. 10.1016/j.wneu.2019.08.005 31404691 10.1016/j.wneu.2019.08.005

[13] Sebök M, Esposito G, van Niftrik CHB et al (2022) Flow augmentation STA-MCA bypass evaluation for patients with acute stroke and unilateral large vessel occlusion: a proposal for an urgent bypass flowchart. J Neurosurg 137:1047–1055. 10.3171/2021.10.JNS21986 34996035 10.3171/2021.10.JNS21986

[14] Zhao M, Charbel FT, Alperin N, Loth F, Clark ME (2000) Improved phase-contrast flow quantification by three-dimensional vessel localization. Magn Reson Imaging 18(6):697–706. 10.1016/s0730-725x(00)00157-0 10930779 10.1016/s0730-725x(00)00157-0

[15] Calderon-Arnulphi M, Amin-Hanjani S, Alaraj A et al (2011) In vivo evaluation of quantitative MR angiography in a canine carotid artery stenosis model. AJNR Am J Neuroradiol 32(8):1552–1559. 10.3174/ajnr.A2546 21835941 10.3174/ajnr.A2546 PMC7964358

[16] Brisman JL (2008) Wingspan stenting of symptomatic extracranial vertebral artery stenosis and perioperative evaluation using quantitative magnetic resonance angiography: report of two cases. Neurosurg Focus 24(2):E14. 10.3171/FOC/2008/24/2/E14 18275290 10.3171/FOC/2008/24/2/E14

[17] Korogi Y, Takahashi M, Mabuchi N et al (1994) Intracranial vascular stenosis and occlusion: diagnostic accuracy of three-dimensional, fourier transform, time-of-flight MR angiography. Radiology 193(1):187–193. 10.1148/radiology.193.1.8090890 8090890 10.1148/radiology.193.1.8090890

[18] Heiserman JE, Drayer BP, Keller PJ, Fram EK (1992) Intracranial vascular stenosis and occlusion: evaluation with three-dimensional time-of-flight MR angiography. Radiology 185(3):667–673. 10.1148/radiology.185.3.1438743 1438743 10.1148/radiology.185.3.1438743

[19] Barlinn K, Alexandrov AV (2011) Vascular imaging in stroke: comparative analysis. Neurotherapeutics 8(3):340–348. 10.1007/s13311-011-0042-4 21691874 10.1007/s13311-011-0042-4 PMC3250265

[20] Ferguson GG, Eliasziw M, Barr HWK et al (1999) The north American symptomatic carotid endarterectomy trial. Stroke 30(9):1751–1758. 10.1161/01.STR.30.9.1751 10471419 10.1161/01.str.30.9.1751

[21] Leng X, Ip HL, Soo Y et al (2013) Interobserver reproducibility of signal intensity ratio on magnetic resonance angiography for hemodynamic impact of intracranial atherosclerosis. J Stroke Cerebrovasc Dis 22(8):e615-619. 10.1016/j.jstrokecerebrovasdis.2013.07.036 24075586 10.1016/j.jstrokecerebrovasdis.2013.07.036 PMC3873834

[22] Miura M, Nakajima M, Fujimoto A, Shiraishi S, Liebeskind DS, Ando Y (2018) Decreased signal intensity ratio on MRA reflects misery perfusion on SPECT in patients with intracranial stenosis. J Neuroimaging 28(2):206–211. 10.1111/jon.12489 29215168 10.1111/jon.12489

[23] Chung JW, Park SH, Kim N et al (2014) Trial of ORG 10172 in Acute stroke treatment (TOAST) classification and vascular territory of ischemic stroke lesions diagnosed by diffusion-weighted imaging. J Am Heart Assoc 3(4):e001119. 10.1161/JAHA.114.001119 25112556 10.1161/JAHA.114.001119 PMC4310410

[24] Hage ZA, Alaraj A, Arnone GD, Charbel FT (2016) Novel imaging approaches to cerebrovascular disease. Transl Res 175:54–75. 10.1016/j.trsl.2016.03.018 27094991 10.1016/j.trsl.2016.03.018

[25] Niesen WD, Weiller C, Sliwka U (2004) Unstable cerebral hemodynamics in carotid artery occlusion and large hemispheric stroke: a cerebral blood flow volume study. J Neuroimaging 14(3):246–250. 10.1111/j.1552-6569.2004.tb00246.x 15228766 10.1177/1051228404265710

[26] Brunberg JA, Frey KA, Horton JA, Deveikis JP, Ross DA, Koeppe RA (1994) [15O]H_2_O positron emission tomography determination of cerebral blood flow during balloon test occlusion of the internal carotid artery. AJNR Am J Neuroradiol 15(4):725–732 8010276 PMC8334202

[27] Sundaram S, Kannoth S, Thomas B, Sarma PS, Sylaja PN (2017) Collateral assessment by CT angiography as a predictor of outcome in symptomatic cervical internal carotid artery occlusion. AJNR Am J Neuroradiol 38(1):52–57. 10.3174/ajnr.A4957 27765736 10.3174/ajnr.A4957 PMC7963674

